# The effect of Tenofovir on renal function among Ugandan adults on long-term antiretroviral therapy: a cross-sectional enrolment analysis

**DOI:** 10.1186/s12981-016-0113-z

**Published:** 2016-08-30

**Authors:** Tino Salome, Ivan Kasamba, Billy Nsubuga Mayanja, Patrick Kazooba, Jackson Were, Pontiano Kaleebu, Paula Munderi, on behalf of the CoLTART study team, Billy N. Mayanja, Judith Nalwadda, Gladys Nakibuuka, Harriet Namugenyi, Patrick Kazooba, Rosemary Lubega, Annet Mugisha, Apophia Tereka, Apuuli Kalyebara, Arthur Namara, Diana Nakitto, Deus Wangi, Fred Nume, George Ssemwanga, Gertrude Nabulime, Gladys Nassuna, Gloria Lubega, Ivan Namakoola, Joseph Lutaakome, Lillian Generous, Lydia Matama, Rosemary Massa, Salome Tino, William Nakahima, Anne A. Kapaata, Brian Magambo, Chris Parry, Frederick Lyagoba, Jamirah Nazziwa, Maria Nannyonjo, Edward Muhigirwa, Faith Wamalugu, Florence Kabajuma, Hope Grania Nakazibwe, Jackson Were, Joan Bwandinga, Juliet Bukenya, Member Zephyrian Kamushaaga, Peter Hughes, Peter Nkurunziza, Priscilla Agatha Balungi, Simon Mukasa, Sureyah Nassimbwa, Tobias Vudriko, William Senyonga, Willyfred Ochola, Annet Nakimbugwe, Catherine Nampewo, Doreen Nambuba, Erima Naphtali, Grace Barigye, Irene Nakamanya, Ivan Kasamba, Jonathan Levin, Joseph Kahwa, Joy Namutebi Matovu, Lillian Namayirira, Ruth Namulindwa Lubega, Sandra Nabalayo, Solomon Kaddu, Paula Munderi

**Affiliations:** 1 MRC/UVRI Uganda Research Unit on AIDS, P.O. Box 49, Entebbe, Uganda; 2 MRC Tropical Epidemiology Group, London School of Hygiene and Tropical Medicine, Keppel Street, London, WC1E 7HT UK; 3 Department of Clinical Research, London School of Hygiene and Tropical Medicine, Keppel Street, London, WC1E 7HT UK

**Keywords:** Antiretroviral therapy, Renal function, Tenofovir, eGFR, HIV

## Abstract

**Background:**

WHO recommends using Tenofovir containing first line antiretroviral therapy (ART), however, Tenofovir has been reported to be associated with renal impairment and dysfunction. We compared renal function among individuals on Tenofovir and those on non-Tenofovir containing ART.

**Methods:**

In a cross-sectional study of HIV-Positive adults on ART, at enrolment into a prospective cohort to study the long-term complications of ART in Uganda, information on biophysical measurements, medical history, clinical examination and renal function tests (RFTs) was collected. Fractional Tubular phosphate reabsorption and estimated glomerular filtration rate (eGFR) were calculated. Mean values of RFTs and proportions with abnormal RFTs were compared between non-Tenofovir containing (Non-TDF) and Tenofovir containing (TDF-ART) ART regimen groups using a general linear regression model. Durations of TDF exposure were also compared.

**Results:**

Between July 2013 and October 2014, we enrolled 953 individuals on ART for 6 or more months, median duration on ART was 9.3 years, 385 (40.4 %) were on non-TDF and 568 (59.6 %) on TDF-ART regimens. The proportion of participants with Proteinuria (>30 mg/dl) was higher among the TDF-ART group than the non-TDF ART group. However, in multivariable analysis, there were no significant differences in the adjusted mean differences of eGFR, serum urea, serum creatinine, fractional tubular reabsorption of phosphate and serum phosphates when patients on TDF-ART were compared with those on non-TDF containing ART. There were no differences in renal function even when different durations on Tenofovir were compared.

**Conclusions:**

We found no differences in renal function among patients on Tenofovir and non-Tenofovir containing ART for almost a decade. Tenofovir based first line ART can therefore safely be initiated even in settings without routine renal function monitoring.

## Background

The WHO recommendation to use Tenofovir in first line antiretroviral therapy (ART) regimens has been widely adopted by ART programmes in Africa including Uganda [1–4]. Due to its cost, Tenofovir had been reserved for use with Protease inhibitors in second line ART regimens. Tenofovir is also used to treat hepatitis B virus infection, and its long intracellular half-life permits a once daily dosing, lower pill burden and facilitates adherence [5, 6]. However, Tenofovir is often associated with renal toxicity, manifesting as a decline in estimated glomerular filtration rate (eGFR), proximal renal tubular dysfunction and acute renal failure especially among patients with risk factors for kidney disease [7–14]. Variations in the onset of Tenofovir associated renal impairment have been reported, in a randomised controlled trial, specific markers of proximal renal tubular function were derailed over 48 weeks in the Tenofovir group [15]. In a Canadian cohort, renal dysfunction was observed in the early years of exposure to Tenofovir but the risk remained minimal beyond 6 years [12]. The median time to develop Tenofovir renal dysfunction was estimated at 154 (15–935) days in a clinical practice in western India [16]. Others have reported that tubular dysfunction or renal failure was diagnosed 6.89 ± 5.51 months after starting Tenofovir [17].

Studies of renal impairment among African patients on ART have reported differing prevalence. In S. Africa, the prevalence of severe (1.3 %) and moderate (13.1 %) renal impairment were reported [18], while in Western Kenya renal insufficiency was reported among 11.5 % of patients on ART [19]. In the DART Trial, there were no significant differences in the incidence of severe decline in eGFR between patients on Tenofovir containing and other ART regimens up to 4 years of therapy. However, patients who initiated Tenofovir based ART with a low eGFR had a greater risk of subsequent severe renal impairment [20]. A review on the renal safety of Tenofovir among HIV-positive patients showed no evidence that Tenofovir use increased the risk of severe proteinuria, hypophosphatemia nor reduced eGFR [21]. However, most of these studies reported on short to mid-term Tenofovir associated renal toxicity, and not the renal dysfunction associated with the long-term use of Tenofovir.

The WHO “Treat all” immediate ART initiation strategy for all HIV-Positive individuals and the use of Tenofovir based first line ART, will result in more HIV infected people getting exposed to Tenofovir [1, 2, 22]. Therefore, since ART is still a life-long intervention, information on the long-term changes in renal function among patients on Tenofovir containing ART is required to inform policy on how such patients should be monitored in the long-term. In this study, we compared renal function among patients who were on or had ever been on Tenofovir with those on non-Tenofovir containing ART regimens.

## Methods

### Study design and settings

This cross-sectional study utilised data collected at enrolment into a prospective clinical cohort established in 2013 to study the Complications of long-term ART among HIV-Positive Ugandan adults (CoLTART). The study settings were the former Development of ART in Africa (DART) study clinic in Entebbe, Central Uganda [23], and the former Rural Clinical Cohort (RCC) study clinic in Kyamulibwa, South western Uganda [24].

### Study population

Study participants were consenting HIV-Positive adults aged 18 years and above, on ART for 6 or more months recruited from two ART cohorts: (1) The DART Trial Cohort, which was established in 2003 as a multisite, randomised clinical trial of monitoring strategy for the management of ART in adults with HIV infection in Africa [23]. After the DART Trial, study participants at the Entebbe site were followed up as a cohort. (2) The RCC which was established in 1990 to study the natural history of HIV infection in a rural Ugandan population [24], and free ART was introduced in 2004. Individuals who were too sick to undergo the study procedures, unable or unwilling to consent were excluded.

### ART regimens

ART regimens were a standard two Nucleoside Reverse Transcriptase Inhibitor (NRTI) and one Non-NRTI first line ART regimen. An alternative first line ART regimen used in the DART Trial cohort was a triple nucleoside (3 NRTI). Second line ART was a combination of one or two NRTI and a ritonavir boosted Protease Inhibitor mainly Lopinavir.

### Study procedures

Potential study participants received the study information, underwent study eligibility assessment and consented for study participation. Data on socio-demographic, socio-economic status, behavioural and medical history of renal disease, hypertension and diabetes mellitus were collected and a clinical examination done. We measured body weight using the Seca digital measuring scale, height using a portable Seca 213 Leicester stadiometer and blood pressure using the Omron M6 comfort automatic blood pressure monitor. Data on patients’ ART history was obtained from the electronic databases of the two former ART cohorts.

### Specimen collection and laboratory methods

Venous blood was collected into three vaccutainer tubes, labelled and transported to the MRC Unit’s Clinical Diagnostic Laboratory Services for laboratory measurements. Renal function tests (blood in a plain serum tube) and fasting blood glucose (blood in a Sodium fluoride tube) were measured using the Clinical chemistry analyser, Cobas Integra 400 plus (Roche Diagnostics). The blood in an EDTA tube was used for: (1) CD4 cell counts measurements using either the FACSCount or FACSCalibur machine (Becton–Dickinson, USA), and (2) Plasma HIV-1 RNA load measured using the COBAS Ampliprep/Taqman V2.0 HIV-1 viral load assay **(**Roche Molecular Diagnostics (RMD), NJ, USA. All participants (apart from women in their menses) provided a fresh midstream urine specimen that was portioned in two plastic centrifuge tubes, one plain for urine creatinine and the other acidified for urine phosphates measurements; and both were measured using the Clinical chemistry analyser, Roche Integra 400 plus (Roche Diagnostics). Proteinuria was measured using a Siemens Multistix 10SG urine dipstick strip test that was read using Clinitek Status Analyzer (Siemens Healthcare Diagnostics).

### Laboratory quality assurance

Standard operating procedures and internal quality measurements ensured internal quality control. The United Kingdom National External Quality Assurance Service (UKNEQAS), College of American Pathologists (CAP) and the Royal College of Pathologists of Australasia (RCPA) were used for External quality Assurance for both haematological and biochemistry assays. Virology Quality Assurance Scheme (Rush University, Chicago, IL) was used for External quality Assurance for virological assays.

### Statistical methods

Data were validated by double-entry on Ms Access systems and STATA 13 (Stata Corporation, College Station, USA) was used for analyses. Renal function was determined using measured renal function tests, and calculated Fractional Tubular reabsorption of phosphate and estimated glomerular filtration rate (eGFR). Different formulae were used to calculate eGFR; (a) the Cockcroft-Gault formulae with and without body surface area (BSA) adjustment, (b) the Modified Diet in Renal Disease (MDRD) formulae with race adjustment (since all our participants were of the African race) and (c) the Chronic Kidney Disease Epidemiology (CKD-Epi) formula. Abnormal measured renal function tests were defined as more than 11.9 mmol/L for serum urea, and more than 109 µmol/L for Serum creatinine. Serum phosphates (mmol/L) was categorised into; Hypophosphataemia (<0.81), normal (0.81–1.5) and Hyperphosphataemia (>1.5), Proteinuria as >30 mg/dl of urine. Fractional Tubular phosphate reabsorption of less than 82 % and values of eGFR less than 60 ml/min/1.73 m^2^ were defined as abnormal. Diabetes mellitus was defined as a measured fasting blood glucose more than or equal to 6.4 mmol/l or history of being told by a professional health worker that one had diabetes mellitus or being on medication for diabetes mellitus prescribed by a professional health worker. Hypertension was defined as measured systolic blood pressure of more than or equal to 140 mmHg or diastolic blood pressure more than or equal to 90 mmHg or any history of being told by a professional health worker that one had raised blood pressure or hypertension or being on medication for raised blood pressure prescribed by a professional health worker.

Renal function outcomes were compared by Tenofovir exposure, as well as duration on Tenofovir and non-Tenofovir containing ART regimens. Tenofovir exposure was categorised as participants on; (1) non-Tenofovir containing ART regimen (Non-TDF), and (2) currently on or ever been on a Tenofovir containing ART regimen (TDF-ART). For the two Tenofovir exposure groups, duration on ART was categorised into; less than 9 and 9 years or more.

Participants’ socio-demographic and economic characteristics, lifestyle and anthropometric as well as clinical, history of diseases and ART exposure were shown by Tenofovir exposure. Mean values of renal function outcomes and proportions with abnormal values were compared by Tenofovir exposure using general linear models and Chi-square tests, respectively. General linear regression models adjusted for age, sex, body mass index, duration on the current ART regimen, alcohol consumption, viral load and CD4 cell counts were used to compare mean values of renal function outcomes across the two Tenofovir exposure groups, as well as duration on Tenofovir and non-Tenofovir containing ART regimen. Adjusted mean differences in renal function outcomes, 95 % confidence intervals for the mean differences, and significance levels are shown.

## Results

### Characteristics of study participants at enrolment

Between July 2013 and January 2014 we enrolled 953 HIV-Positive individuals who had been on ART for 6 or more months, 628 (65.9 %) were females and 90.7 % were aged 35 years and above. The median duration on ART was 9.3 years and most participants (68.6 %) had been on ART for 9 or more years, 84.2 % had suppressed viral loads of less than 1000 copies/ml and 65.4 % had CD4 cell counts of 351 cells/µl or higher. The number of patients on non-Tenofovir containing ART (Non-TDF) was 385 (40.4 %) while 568 (59.6 %) were or had been on a Tenofovir containing ART (TDF-ART) (Table 1).

**Table 1:** Characteristics of participants who had been on ART for more than 6 months at enrolment by Tenofovir Disoproxil Fumarate (TDF) exposure

Characteristics	All participants n (%)	TDF exposure	
		Non-TDF ART n (%)	TDF ART n (%)
All^a^	953	385 (40.4)	568 (59.6)
*Socio-demographic/economic*			
Age (years)			
18–34	89 (9.3)	45 (11.7)	44 (7.7)
35–49	578 (60.7)	222 (57.7)	356 (62.7)
50+	286 (30.0)	118 (30.6)	168 (29.6)
Sex			
Females	628 (65.9)	250 (64.9)	378 (66.5)
Males	325 (34.1)	135 (35.1)	190 (33.5)
SES score tertile^b^			
Low	403 (42.3)	165 (42.9)	238 (41.9)
Middle	359 (37.7)	144 (37.4)	215 (37.9)
High	186 (19.5)	76 (19.7)	110 (19.4)
*Lifestyle, anthropometric*			
Tobacco consumption			
Never	791 (83.0)	305 (79.2)	486 (85.6)
Ex-smoker	93 (9.8)	39 (10.1)	54 (9.5)
Current	68 (7.1)	41 (10.6)	27 (4.8)
Alcohol consumption			
Never	358 (37.6)	141 (36.6)	217 (38.2)
Ever >1 month	352 (36.9)	135 (35.1)	217 (38.2)
Within <1 month	234 (24.6)	108 (28.1)	126 (22.2)
Body Mass Index (kg/m^2^)			
<18.5	91 (9.5)	30 (7.8)	61 (10.7)
18.5–24.9	591 (62.0)	263 (68.3)	328 (57.7)
25.0-29.9	192 (20.1)	69 (17.9)	123 (21.7)
≥30	60 (6.3)	18 (4.7)	42 (7.4)
*Clinical, disease history, art*			
Hypertension			
Normal	722 (75.8)	299 (77.7)	423 (74.5)
Hypertensive	220 (23.1)	83 (21.6)	137 (24.1)
Glucose (mmol/lL)/history			
Desirable (≤6.4 mmol/l)	912 (95.7)	375 (97.4)	537 (94.5)
High (>6.4 mmol/l)	33 (3.5)	10 (2.6)	23 (4.0)
Renal disease history			
No renal disease	946 (99.3)	383 (99.5)	563 (99.1)
Known renal disease	6 (0.6)	2 (0.5)	4 (0.7)
ART regimen type/class			
First line ART			
2NRTIs/NNRTI	481 (50.5)	312 (81.0)	169 (29.8)
3NRTIs	237 (24.9)	41 (10.6)	196 (34.5)
Second line ART (with boosted PI)	235 (24.7)	32 (8.3)	203 (35.7)
Cotrimoxazole use			
Yes	570 (59.8)	161 (41.8)	409 (72.0)
No	383 (40.2)	224 (58.2)	159 (28.0)
Total duration on ART (years)			
0.5 to <5	175 (18.4)	138 (35.8)	37 (6.5)
5 to <9	124 (13.0)	79 (20.5)	45 (7.9)
9+	654 (68.6)	168 (43.6)	486 (85.6)
HIV-1 viral load (copies/ml)			
<1000	802 (84.2)	346 (89.9)	456 (80.3)
1000–9999	48 (5.0)	13 (3.4)	35 (6.2)
10,000+	71 (7.5)	15 (3.9)	56 (9.9)
CD4 cell counts (cells/µl)			
≤350	263 (27.6)	102 (26.5)	161 (28.3)
351–500	274 (28.8)	120 (31.2)	154 (27.1)
501+	349 (36.6)	149 (38.7)	200 (35.2)

^a^Percentages in brackets show percentage of TDF or non-TDF containing ART over all patients on ART

^b^SES is socio-economic status index calculated over the total items owned. Hypertension defined as SBP ≥140 mmHg or DBP ≥90 or any history of hypertension

### Glomerular function

The overall proportions with renal dysfunction or renal failure and abnormal renal function assessment parameters were low among our study participants. However, in the unadjusted analysis, the mean eGFR was lower among the TDF-ART group than the non-TDF ART group using the CKD-Epi formula (P = 0.001) and MDRD formula with race adjustment (P = 0.008), but we found no differences in the mean eGFR calculated using the Cockcroft-Gault formulae (both with and without body surface area adjustment). Using all the four eGFR formulae, although the proportions of participants with abnormal eGFR (<60 ml/min/1.73 m^2^) were higher among the TDF-ART than the non-TDF ART group, these differences were not significant. Overall, participants in the TDF ART group had higher mean serum urea levels (P = 0.001) and higher mean serum creatinine levels (P = 0.023) than those in the non-TDF ART group, however the numbers with abnormal serum urea and creatinine levels were small (Table 2). In multivariable analysis, we found no significant differences in the adjusted mean differences in eGFR between TDF-ART and non-TDF ART groups using any of the formulae. We still found no significant differences in the adjusted mean differences of eGFR when different durations on different TDF exposure ART regimes were compared (Table 3).

**Table 2:** Renal dysfunction or failure and mean values of renal function assessment parameters among patients with and without Tenofovir Disoproxil Fumarate containing ART regimen

Renal function assessment parameter	All participants n (%)	TDF exposure		P value*
		Non-TDF ART n (%)	TDF ART n (%)	
All	*953*	*385* (*40.4)*	*568* (*59.6*)	
eGFR (Cockcroft-Gault, adj for BSA), mean (SD)	*108.6* (*37.0*)	*110.0* (*36.0*)	*107.3* (*36.8*)	*0.197*
Normal	901 (97.0)	371 (97.6)	530 (96.5)	0.338
Renal dysfunction/failure	28 (3.0)	9 (2.4)	19 (3.5)	
eGFR (Cockcroft-Gault, without BSA adj), mean (SD)	*104.9* (*68.4*)	*103.6* (*58.9*)	*104.6* (*71.3*)	*0.933*
Normal	874 (93.9)	363 (95.3)	511 (92.9)	0.139
Renal dysfunction/failure	57 (6.1)	18 (4.7)	39 (7.1)	
eGFR (CKD-Epi), mean (SD)	*102.9* (*18.0*)	*105.3* (*17.5*)	*101.7* (*18.2*)	*0.001*
Normal	929 (98.1)	378 (98.2)	551 (98.0)	0.878
Renal dysfunction/failure	18 (1.9)	7 (1.8)	11 (2.0)	
eGFR (MDRD with race), mean(SD)	*131.7* (*43.8*)	*135.9* (*47.0*)	*128.9* (*40.2*)	*0.008*
Normal	935 (98.7)	382 (99.2)	553 (98.4)	0.267
Renal dysfunction/failure	12 (1.3)	3 (0.8)	9 (1.6)	
Fractional Tubular PO4 reabsorption %, mean (SD)	*92.1* (*6.1*)	*92.7* (*5.7*)	*91.7* (*6.3*)	*0.047*
Normal	734 (95.7)	325 (95.3)	409 (96.0)	0.634
Abnormal Fractional Tubular PO4 reabsorption	33 (4.3)	16 (4.7)	17 (4.0)	
Proteinuria on dipstick (mg/dl)				
Negative	646 (69.5)	298 (78.6)	348 (63.2)	<0.001
Trace (1–30)	120 (12.9)	38 (10.0)	82 (14.9)	
Positive (30+)	164 (17.6)	43 (11.3)	121 (22.0)	
Serum urea (mmol/L), mean (SD)	*3.1* (*1.3*)	*3.0* (*1.2*)	*3.2* (*1.4*)	*0.001*
Desirable	944 (99.8)	384 (100.0)	560 (99.6)	0.242
Abnormal (>11.9)	2 (0.2)	0 (0.0)	2 (0.4)	
Serum creatinine (µmol/L), mean (SD)	*65.5* (*20.3*)	*64.0* (*18.5*)	*66.8 *(*20.8*)	*0.023*
Desirable	930 (98.2)	381 (99.0)	549 (97.7)	0.147
Abnormal (>109)	17 (1.8)	4 (1.0)	13 (2.3)	
Serum phosphates (mmol/L), mean (SD)	*1.0* (*0.3*)	*1.0* (*0.2*)	*1.1* (*0.3*)	*0.023*
Hypophosphataemia (<0.81)	122 (13.1)	64 (16.6)	58 (10.6)	0.012
Normal (0.81–1.5)	799 (85.5)	318 (82.6)	481 (87.6)	
Hyperphosphataemia (>1.5)	13 (1.4)	3 (0.8)	10 (1.8)	

*BSA adj* body surface area adjustment

* P value showing evidence for mean differences in estimated parameters by TDF exposure (in italics) and Chi-square test P value (not in italics). Renal dysfunction/failure defined as eGFR: <60 ml/min/1.73 m^2^. Some missing eGFR values due to missing some anthropometric measures used to calculate eGFR e.g. patients who could not stand upright because of being lame

**Table 3:** Adjusted mean differences in renal function assessment parameters among patients on TDF and without TDF containing ART

Renal function assessment parameter	Tenofovir (TDF) ART exposure		duration on TDF or non-TDF containing ART			
	TDF versus non-TDF	P value*	Non-TDF 9 + y versus non-TDF < 9y	TDF < 9y versus non-TDF < 9y	TDF 9 + y versus non-TDF < 9y	P value*
	aMD (95 % CI)^a^		aMD (95 % CI)^a^	aMD (95 % CI)^a^	aMD (95 % CI)^a^	
eGFR (Cockcroft-Gault, with BSA adj)	−2.26 (−8.46 to 3.94)	0.472	−2.97 (−13.97 to 8.03)	3.79 (−6.68 to 14.26)	−7.39 (−18.10 to 3.32)	0.200
eGFR (Cockcroft-Gault, without BSA adj)	−2.79 (−14.50 to 8.93)	0.638	−4.40 (−25.21 to 16.42)	1.30 (−18.50 to 21.11)	−8.43 (−28.70 to 11.84)	0.778
eGFR (CKD-Epi)	−0.97 (−3.85 to 1.91)	0.507	−0.32 (−5.44 to 4.79)	2.92 (−1.95 to 7.80)	−2.83 (−7.82 to 2.16)	0.114
eGFR (MDRD with race adj)	−0.30 (−7.58 to 6.98)	0.935	−2.68 (−15.61 to 10.25)	7.26 (−5.05 to 19.57)	−5.77 (−18.37 to 6.83)	0.248
Fractional tubular PO4 reabsorption, % (SD)	−0.47 (−1.54 to 0.61)	0.390	−0.16 (−2.01 to 1.68)	−0.86 (−2.63 to 0.91)	−0.44 (−2.24 to 1.36)	0.786
Urea BUN	0.07 (−0.14 to 0.29)	0.503	−0.05 (−0.43 to 0.33)	0.28 (−0.08 to 0.65)	−0.05 (−0.43 to 0.32)	0.302
Serum creatinine (µmol/L)	2.47 (−0.86 to 5.81)	0.143	−2.51 (−8.44 to 3.42)	−1.53 (−7.17 to 4.12)	1.81 (−3.97 to 7.59)	0.161
Serum phosphates (mmol/L)	0.00 (−0.04 to 0.05)	0.913	−0.01 (−0.09 to 0.08)	0.03 (−0.05 to 0.11)	−0.01 (−0.09 to 0.07)	0.823

*BUN* blood urea nitrogen, *BSA adj* body surface area adjustment, *race adj* race adjustment

* P value showing evidence for adjusted mean differences in renal function parameters by ART regimen type, and duration

^a^aMD (95 % CI) is adjusted mean difference with 95 % Confidence Interval, adjusted for site, duration on ART for those on non-TDF and duration on TDF among those on TDF, tobacco consumption, socio-economic status index, viral load, CD4 cell counts, hypertension and history of diabetes mellitus or high glucose levels

### Renal tubular function

In the unadjusted analysis, the mean fractional tubular phosphate reabsorption was lower in the TDF-ART group than the Non-TDF ART group [mean (SD); 91.7 (6.3) vs 92.7 (5.7), P = 0.047]. However, we had few patients with abnormal fractional tubular phosphate reabsorption mean values and no significant differences were found between the two comparison groups. The proportion of patients with proteinuria (30 mg/dl or more) was higher in the TDF-ART group [n = 121 (22.0 %)] than in the Non-TDF ART group [n = 43 (11.3 %)], P < 0.001. The mean serum phosphates was slightly higher in the TDF-ART group (1.1 mmol/L, SD 0.3), than in the Non-TDF ART group (1.0 mmol/L, SD 0.2), P = 0.023, but the proportions of patients with hypophosphataemia were higher among the Non-TDF ART [n = 64 (16.6 %)] than the TDF-ART group [n = 58 (10.6 %)], P = 0.012 (Table 2). In multivariable analysis, when the TDF-ART and Non-TDF ART groups were compared, we found no significant differences in the adjusted mean differences in the fractional tubular reabsorption of phosphate and serum phosphates. We still found no significant differences in the adjusted mean differences of fractional tubular reabsorption of phosphate and serum phosphates when durations on the two TDF exposure ART groups were compared (Table 3).

## Discussion

In this cross-sectional study among HIV-Positive individuals on antiretroviral therapy for a median of 9.3 years, we found no differences in renal function (eGFR and fractional tubular phosphate reabsorption) among patients on Tenofovir and Non-Tenofovir containing ART regimens. Even with different durations on ART, we still found no significant differences in renal function among patients by TDF exposure ART group. These findings might allay concerns raised about the renal toxicity of Tenofovir which is recommended for initiating ART by the WHO, and has been widely adopted by ART programmes in many resource limited countries including Uganda [1–4].

Previous studies have documented the association between Tenofovir and renal dysfunction, leading to glomerular and proximal renal tubular damage and acute renal failure [8–14]. However, we found no differences in glomerular function between patients who were on Tenofovir containing ART and those who were on Non-Tenofovir containing ART. Other studies have similarly reported no or minimal reductions in glomerular filtration rate among patients on Tenofovir [17, 21, 25]. Similarly, a 10 year follow-up cohort study of HIV-positive patients, reported that the quantified loss in eGFR attributable to Tenofovir was relatively modest after many years of exposure [12]. Since the majority of our patients had been on ART for more than 9 years, our failure to find an association between renal dysfunction and duration on Tenofovir containing ART can be explained by previous studies that reported that the loss in eGFR attributable to Tenofovir seemed to occur during the first years of exposure between 0.5 and 31.2 months and stabilized after that [16, 17, 25–28]. Post marketing studies reported advanced age, low body weight, comorbidities such as diabetes mellitus, hypertension, as some of the risk factors for Tenofovir induced renal dysfunction [27, 29, 30]. Therefore, our finding of no differences in renal function between the Tenofovir and non-Tenofovir containing ART regimens might be explained by the small proportions of individuals aged above 50 years, low BMI < 18.5 kg/m^2^, with high blood sugar, hypertension and known renal disease. One study reported that the odds of developing significant renal dysfunction was 3.7 times higher among patients receiving Tenofovir and ritonavir-boosted Protease inhibitor (boosted-PI) regimens than those receiving Tenofovir and non-nucleoside reverse transcriptase inhibitor-based therapy [30]. This might explain our finding of no increase in renal dysfunction among patients on Tenofovir, as in our study, only 24.7 % on were on second line ART containing Tenofovir and a boosted PI.

We observed a higher proportion of patients with proteinuria (>30 mg/dl) among patients on Tenofovir containing ART than those on Non-Tenofovir ART regimens. The significant presence of proteinuria among the TDF-ART group when compared to the Non-TDF ART group can be explained by the fact that proteinuria is a presentation of subclinical proximal tubular dysfunction without glomerular function impairment [11, 31]. Our findings are comparable to reports from a retrospective cohort of HIV infected US veterans where Tenofovir use was found to be associated with an increased risk of proteinuria and chronic kidney disease [32]. Although in univariate analysis we observed a lower mean fractional tubular phosphate reabsorption among patients on TDF-ART than those on Non-TDF ART, the differences were of borderline significance and disappeared in multivariable analysis. We found that the proportion of patients with hypophosphataemia was higher among patients on Non-TDF ART than those on TDF-ART regimens. However, a study that assessed renal tubular dysfunction during long-term Adefovir or Tenofovir therapy in chronic Hepatitis B infection reported that 7 (14 %) out of 51 patients developed persistent hypophosphataemia, with urinary phosphate wasting and a low (<82 %) estimated fractional tubular reabsorption of phosphate, but their sample size was small and they lacked a comparison group [33].

Our study strengths included the availability of a comparison group of patients on non-Tenofovir containing ART which enabled control for residual confounding. We also assessed glomerular function as well as proximal renal tubular function since Tenofovir has been reported to affect both renal functions. The long duration of our patients on ART, allowed us to document the effect of long-term Tenofovir exposure. Most previous reports were of shorter duration on Tenofovir, because in sub Saharan Africa, due to its cost, Tenofovir was previously reserved for second line ART.

We acknowledge some limitations that might have affected our study findings; a small number of patients (50) had discontinued Tenofovir but we did not consider the period since Tenofovir was discontinued and the reasons why it was discontinued. If Tenofovir was stopped due to Tenofovir induced renal dysfunction, this might have biased our results especially if the duration since Tenofovir stoppage was short. Although none of our participants was known to be using nephrotoxic medications like amphotericin, and we did not collect data on previous use of such medications, some residual confounding is still possible. Since samples collected for HBV and HCV testing have not yet been tested, we did not assess the effect of these co-infections on renal function among our study participants. Due to the cross-sectional study design of our study, assessing single point proteinuria instead of the more clinically significant persistent proteinuria might have biased our findings. We could not compare our findings with the pre-ART renal function because in our setting, routine renal function test measurements are not done prior to ART initiation. Furthermore, because our patients were former research study participants with close monitoring, our findings may not be generalizable to the general population of HIV-infected patients who receive ART in the general HIV care programmes.

## Conclusions

In conclusion, we found no differences in eGFR and fractional tubular phosphate reabsorption among patients on Tenofovir and non-Tenofovir containing ART after a median of 9.3 years on ART. Since in most resource limited settings, routine renal function testing prior to ART initiation and monitoring during treatment is not always feasible, our results are reassuring as ART programmes adopt the WHO recommendation to use Tenofovir in first line ART regimens. Therefore, the WHO recommended Tenofovir based first line ART can safely be initiated even in settings without routine renal function monitoring. However, the higher proteinuria among patients on Tenofovir containing ART in the absence of a corresponding higher impairment of fractional tubular phosphate reabsorption suggests a subclinical proximal tubular dysfunction, therefore targeted renal function monitoring for specific patients should be undertaken when clinically indicated. Further evaluation of renal function among patients on Tenofovir beyond 10 years is advised.

## Abbreviations

ART: antiretroviral therapy
BSA: body surface area
BUN: blood urea nitrogen
CAP: College of American Pathologists
CKD-Epi: chronic kidney disease epidemiology
CoLTART: complications of long-term antiretroviral therapy
DART: development of antiretroviral therapy in Africa
eGFR: estimated glomerular filtration rate
HIV: human immunodeficiency virus
MDRD: modified diet in renal disease
NRTI: nucleoside reverse transcriptase inhibitors
NNRTI: non nucleoside reverse transcriptase inhibitors
RFTs: renal function tests
PI: protease inhibitors
PO4: phosphate
RCC: rural clinical cohort
RCPA: Royal College of Pathologists of Australasia
RMD: roche molecular diagnostics
SD: standard deviation
SES: social economic status
TDF: Tenofovir Disoproxil Fumarate
UKNEQAS: United Kingdom National External Quality Assurance Service
WHO: World Health Organisation
